# Hypertensive Disorders of Pregnancy: A Window into Breastfeeding Outcomes in Varied Healthcare Systems

**DOI:** 10.3390/nu16193239

**Published:** 2024-09-25

**Authors:** Jimi Francis, Elizabeth Gelner, Darby Dickton

**Affiliations:** 1 Department of Kinesiology, College for Health, Community, and Policy, University of Texas at San Antonio, San Antonio, TX 78249, USA; 2 Brooke Army Medical Center, San Antonio, TX 78249, USA; 3 Foundation for Maternal, Infant, and Lactation Knowledge, San Antonio, TX 78249, USA; ddickton@foundation4milk.org

**Keywords:** hypertensive disorders of pregnancy, gestational hypertension, breastfeeding initiation, hospital policy, preterm infants

## Abstract

Varied hospital systems demonstrate diverse and often very different approaches to patient care. This may best be analyzed by looking at specific disorders and outcomes in a population with these disorders. As one such lens, hypertensive disorders of pregnancy (HDPs) continue to pose a severe health risk for mothers and infants, and breastfeeding outcomes play a crucial role in determining long-term maternal and fetal health. This pilot study investigated breastfeeding outcomes in two hospitals, as representatives for differing healthcare systems, among patients diagnosed with HDPs. Data were collected for 12 months at two hospitals, one private and one military, on 729 patients diagnosed with HDPs. Data were analyzed for infant maturity at birth and breastfeeding outcomes. Most participants (83.2%) stated their intention to breastfeed for the first six months of life. By hospital discharge, only 56% (*p* = 0.0001) of the private hospital participants were breastfeeding compared to 65% of the military hospital participants. In addition, while 69% of infants were born before term, more infants were born before term at the private hospital (71%) than at the military hospital (65%), with 37% (private) and 42% (military) of preterm infants breastfeeding at hospital discharge. Significant differences existed between these two hospital systems in the number of those initiating breastfeeding and breastfeeding at hospital discharge. The military hospital was more successful in assisting these high-risk women in meeting their breastfeeding intentions. Changes in hospital practices, such as metrics and incentivization, focusing on breastfeeding support, could improve the rate of breastfeeding at hospital discharge and impact long-term health outcomes.

## 1. Introduction

Hospital systems are increasingly far-reaching and use a network of algorithms to guide a system-wide standardized approach to medical practices. Medical science has made significant progress in every aspect, including maternal [1] and infant care [2]. However, hypertensive disorders of pregnancy (HDPs) continue to pose a severe health risk for both mothers and infants before and after childbirth [3]. Complications of these disorders have a long-standing history and are considered by some as “unresolved and unpreventable [4]”. In contrast, others posit that HDPs are “the leading preventable cause of premature death [5]”. HDPs are a group of conditions that occur during pregnancy and are characterized mainly by high blood pressure (hypertension) [6]. HDPs is an umbrella term that includes chronic hypertension, gestational hypertension, preeclampsia, and eclampsia [7]. These diseases are an effective vehicle for assessing the difference in outcomes between two hospital systems.

HDPs affect between 3% to 14% of pregnant women worldwide based on data updated in 2016 [8], and this percentage has increased in the United States between 2017 and 2019 to 16% [9]. HDPs can have severe consequences for both mother and fetus. High blood pressure during pregnancy can reduce blood [10] flow to the placenta, leading to insufficient oxygen and nutrients for the developing fetus. This can result in premature birth or low birth weight and may require induction of labor or cesarean delivery. After pregnancy, mothers remain at high risk of hypertension and cardiovascular disease [11], while both mother and baby are at increased risk of ischemic heart disease and stroke [12]. Prematurity can also lead to long-term health problems for the infant, including breathing difficulties, infections, cognitive impairment, and retinopathy [13]. Even if the fetus reaches full term, there is a risk of complications, such as stillbirth [10], neonatal death [13], or breastfeeding challenges [14].

Breastfeeding is widely recognized as essential for promoting optimal health and development in infants and mothers [15,16,17]. The numerous benefits of breastfeeding are well established, yet exclusive breastfeeding rates and duration remain below the levels recommended worldwide [18].

It has been theorized that some negative health consequences for mothers and infants can be moderated through breastfeeding, with mother’s milk playing a fundamental role in health [19,20]. Research has shown maternal and infant protective health associations between breastfeeding and HDPs, both before and after pregnancy. Countouris et al. reported that overweight women who had previously breastfed had lower systolic blood pressure with HDPs if they had previously breastfed for six months or more [21]. Many studies have shown a dose–response relationship between breastfeeding and hypertension later in life [22,23,24]. Among children who have been breastfed, those with a longer duration of breastfeeding have shown an associated decreased risk of hypertension in adulthood [25]. Given the complications during pregnancy and the interventions that may be needed during birth, it is reasonable to conclude that hospital policies regarding the management of HDPs could impact breastfeeding.

Hospital policies and systems play a pivotal role in shaping the early breastfeeding experiences of new mothers [26]. The policies implemented within healthcare institutions can act as catalysts or barriers to successful breastfeeding initiation and continuation [27]. Understanding the intricate interplay between hospital policies and breastfeeding rates when faced with health complications is crucial in improving breastfeeding outcomes [28]. Concerns about HDPs and breastfeeding exist in all mother/baby units, but how each unit addresses these concerns may drastically affect the outcomes.

Using two hospitals with drastically different approaches to prenatal healthcare access and hospital protocols regarding breastfeeding support, we used hypertension disorders of pregnancy to establish objective markers to assess the outcomes between the two systems. One hospital was used to exemplify the more common private hospital healthcare options, while the other was used as a marker for the healthcare system as managed by the military. Would two hospitals show statistically similar outcomes for breastfeeding among patients with HDPs, given their vastly different resources and systems, despite the same accepted standard of care practices?

## 2. Materials and Methods

### 2.1. Study Design

This observational pilot study was cross-sectional, with data collected from two hospital locations for 12 months ending at the end of 2020. The data reported here compare all the patients diagnosed with hypertensive disorders of pregnancy admitted for infant delivery at two hospitals. Additional data analyses for each hospital dataset are reported elsewhere [29,30]. The Institutional Review Boards for the participating hospitals and the University of Texas identified the study as exempt from informed consent, as any identifying information was de-identified following the human subjects’ research policy before the researchers received the data.

### 2.2. Setting

In this study, the private hospital (PH) is a not-for-profit private health system that provides community care in underserved communities. During the study, the PH had 3600 personnel maintaining a full-service facility providing inpatient, outpatient, and emergency care in a 460-bed facility with over 2700 births yearly. The PH had diverse care pathways and protocols across care providers, as it needs long-term continuity of care for patients due to the transient population. In contrast, this study’s military hospital (MH) had 1400 personnel maintaining a full-service, 65-bed hospital providing inpatient, outpatient, and emergency care for military members and their dependents, with 1029 births in the year studied. The military system was extensively integrated across care venues with robust continuity of care pathways for patients. For example, electronic medical record systems across the OB and pediatric offices at military facilities are integrated with physicians, often in close contact. In addition, the military care algorithms were standardized among providers, meaning that care across all patients is relatively consistent. Both hospitals used the Joint Commission’s Perinatal Care Performance Standards for breastfeeding, PC-05 [31].

### 2.3. Participants

The participants encompassed all of the patients with diagnoses of hypertensive disorders of pregnancy based on diagnosis codes at hospital admittance for the birthing of the infant at the two hospital settings.

Both hospital Institutional Review Boards approved the research plan with a designation of unregulated research, as hospital staff de-identified the data before the researchers received them. The University of Texas Institutional Review Board served as the oversight body.

### 2.4. Variable Definitions and Statistical Analysis

The hospital staff collected all variable data from patient charts onto a spreadsheet provided to the research team. HDPs were determined by the hospital diagnosis codes and grouped into three categories based on the similarity of the diagnosis codes: chronic hypertension (CH), gestational hypertension (GH), and a combined group of preeclampsia and eclampsia (PREE). For this study, infant maturity was defined as preterm (PRT), less than 37 weeks of gestation; early term (ET), 37–38 weeks and six days of gestation; term (T), between 39 and 40 weeks and six days of gestation; and late-term (LT), equal to or more than 41 weeks of gestation.

Feeding at discharge was defined using the WHO definition of exclusive breastfeeding (BF) (exclusive feeding directly from the breast or human milk from a bottle, cup, or syringe with no other solids or liquids) [32], formula feeding (FF), or mixed feeding of human milk and formula (MF).

Statistical analysis was performed using IBM SPSS, version 28.0.0.0 (190), made in Armonk, NY, USA. The variables of interest were evaluated using descriptive statistics. Cross-tabulation analysis was conducted for categorical data analysis. Data were analyzed according to the HDP category, infant maturity at birth, breastfeeding intention upon admission, breastfeeding initiation in the first hour of life, and feeding modality at discharge. In addition, a cross-tabulation analysis was conducted on the relationship between HDPs and infant maturity, feeding status at discharge, and hospital locations. Frequencies and descriptive statistics are reported.

## 3. Results

### 3.1. Diagnosis by Hospital Location

The total number of participants was 714, with 74% (*n* = 529) in the PH group and 26% (*n* = 185) in the MH group. For the PH group, 19% of the birthing dyads (*n* = 529 of 2763), and for MH, 18% (*n* = 185 of 1029) of the birthing dyads were positive for HDPs, which was not significantly different between the facilities. Evaluating the categories of HDPs based on diagnosis codes at both hospitals, the analysis revealed significant differences, with a Pearson Chi-square of 67.77 (*p* = 0.001) between the hospitals for the category of HDPs, with CH at 2.1%, GH at 78.6%, and PREE at 19.3% in the PH compared to the MH, with 21.10% CH, 44% GH, and 35% PREE, respectively (Table 1). In addition, the percentage of participants with chronic hypertension was significantly lower in the PH group than in the MH group.

HDPs were significantly associated with preterm birth, as grouped into CH, GH, or PREE, with a Pearson Chi-square of 39.16 (*p* = 0.001). Of the 714 mother/infant dyads, 69.2% were PRT or ET, and only 30.8% were T or LT. When identified by hospital location, the PH group had 70.5% preterm or early-term HDP deliveries, with 178 of 529 (34%) infants delivered preterm and 195 of 529 (37%) infants delivered ET. In contrast, the MH group had 65% delivered either preterm or early term, with 29% delivered T or LT. The MH had 3.4% preterm, 61.6% ET, and 34.6% T or LT infants.

### 3.2. Infant Maturity by Hospital Location

Infant maturity differences at birth were statistically significant between the locations (Pearson Chi-square 46.26, *p* < 0.001). The rate of preterm births at the PH was 34% of the 529 births, compared to 4% of PRT births at the MH, with 26% of the total (714) born preterm. ET births at the PH were 37%, and at the MH, 61%. At the PH, the rate for infants born before term was 71%, and at the MH, the rate for infants born before term was 65%. The PH rate for T births was 28%, compared to 32% at the MH. The rate of infants born in the LT at the PH was 1.7%, compared to 2.2% at the MH, which was not significantly different. In the combination of term and late-term infants, the rate of infants born at or after term (*n* = 220) was 25%. Separately, the rate of infants born term or later was 30% in the PH and 35% in the MH. The numbers and rates of infant maturity according to hospital location are shown in Table 2.

### 3.3. Breastfeeding Intention

**Figure 1:**
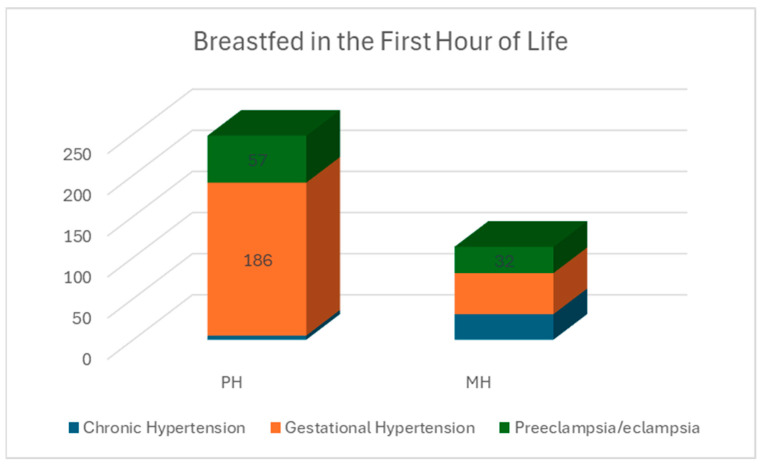
Infants that initiated breastfeeding in the first hour of life by hospital.

The intention to breastfeed was similar between the hospitals. The percentage of those stating they intended to breastfeed was slightly lower in the PH group, at 83% (439 out of 529), than in the MH group, at 84% (155 out of 185). However, it was not statistically significant (Fisher’s Exact *t*-test *p* = 0.451). Across all the participants, in the first hour of life, 51% (*n* = 361) initiated breastfeeding, while 49% (*n* = 353) did not. When evaluated by hospital and HDP category, initiation in the first hour of life differed. In the PH, 47% of the participants breastfed in the first hour of life compared to 61% at the MH, which was significant (Pearson Chi-square 11.06 *p* = 0.002). Based on the HDP category, at the PH, of those who initiated breastfeeding in the first hour of life, 2% had CH, 75% had GH, and 23% had PREE. Of those who breastfed in the first hour at the MH, 27% had CH, 44% had GH, and 28% had PREE, as shown in Figure 1.

### 3.4. Feeding Modality at Hospital Discharge

**Figure 2:**
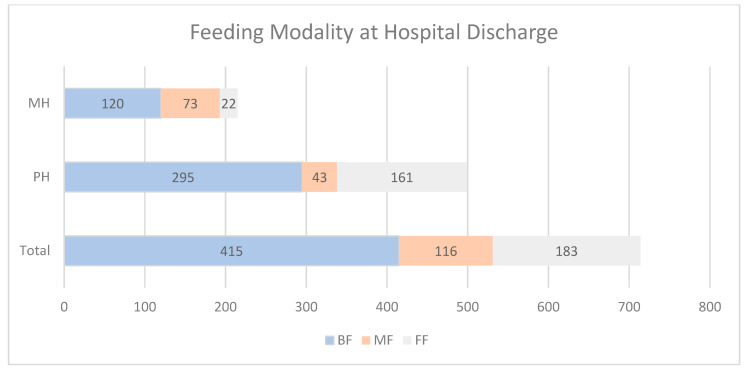
Type of Feeding at Hospital Discharge for Military Hospital (MH) versus Private Hospital (PH), where BF = Breastfeeding, MF = Mixed Feeding, and FF = Formula Feeding.

Overall, 58% (415 of 714) of infants were BF at hospital discharge, with 26% (183 of 714) FF and 16% (116 of 714) MF. The rate of BF at hospital discharge was significantly different between hospitals, with a Pearson Chi-square of 32.579 (*p* = 0.001). At the PH, 56% (295 of 524) were BF at hospital discharge, while at the MH, 65% (120 of 185) were BF at discharge, with a Pearson Chi-square of 32.58, *p* < 0.001 (See Figure 2).

#### Infant Maturity by Hospital Location by Diagnosis and Feeding Modality at Hospital Discharge

When categorized by HDP, for the CH category at the PH, 1% (5 of 524) were BF, 1% were FF, and 1% were MF. At the MH, 17% (31 of 185) were BF, 1% were FF, and 3% were MF. Among all patients with GH, 38% (267 of 714) were BF, 21% were FF, and 12% of the infants were MF at hospital discharge. Of those participants with the PREE category, 16% (112 of 709) were BF, 4% (30 of 709) were FF, and 3% (24 of 709) of the infants were MF at hospital discharge. At the PH, for patients with PREE, 14% (73 of 524) were BF, 4% were FF, and 2% were MF. At the MH, 21% (39 of 185) were BF, 5% were FF, and 9% were MF, as shown in Table 3.

### 3.5. Strengths and Limitations

A significant strength of the study was that some of the authors served as care providers at the facilities. They were familiar with the procedures and policies of the institutions involved and understood the differences between hospital practices.

This study looked only at one hospital representative from each of the two different hospital systems, limiting the confidence that the results could be applied to all hospitals. Although inference of common improvement strategies, such as metrics assessing morbidity prevention, can be promising, system-wide improvement requires analyzing different hospital systems, in this case, military versus private. Future studies could examine outcomes between different private hospital systems, such as those wrapped into insurance programs, such as the Kaiser system, and profit-based systems.

## 4. Discussion

A single hospital cannot generally dictate the outcomes for whole hospital systems, but these two hospitals serve as a landmark to initiate conversations about potential systematic changes that could improve outcomes. The intention to breastfeed did not differ between the military and private hospital settings, which speaks well of programs aimed at increasing breastfeeding initiation rates. Unfortunately, the rate of BF at hospital discharge was lower in the PH (47%) than in the MH (65%) and far below breastfeeding intentions for both locations, indicating that the MH staff was more successful than the PH staff in assisting women in meeting their breastfeeding intentions by hospital discharge in the face of adversity with an HDP diagnosis. Given the authors’ experience with public, private, and military hospitals, the statistical differences between CH and GH at the two facilities could partly be accounted for by differing methods of diagnosing these two diseases, particularly given that diagnosis relies on the ability to look back at a patient’s history reliably, and there is continuity of care provided within the military. The military system would likely have had regular access to baseline health data to differentiate between CH and GH. Nevertheless, even if the CH and GH categories were tallied together, the PH had a 16% higher rate of hypertension. Prenatal care access is readily available and encouraged, in some cases mandated, amongst the population at the MH. Not only will prenatal care improve the ability of providers to differentiate between CH and GH, but it will also initiate a care algorithm to ensure improved outcomes in high-risk patients. As MH systems also have invested interest in preventative outcome systems, due to their sponsorship of the medical bills accumulated from complications, they often have systems built in to encourage optimized outcomes. It is not uncommon for military hospitals to require educational briefings or classes to address health concerns more economically, with each patient being briefed individually by their provider.

Despite the significant increase in PREE observed in the MH, their breastfeeding outcomes were better than those seen in the PH. While these data show an interesting correlation, it is hard to determine whether the regimentation of care at the MH has “improved those rates” or if other factors at the community hospital hinder similar outcomes, specifically the availability of long-term continuous care. This finding again speaks to the interest in preventative-focused medicine practices at the hospital system level. As the guarantor for the billing, MH systems benefit financially from encouraging breastfeeding, which will lead to healthier outcomes. In contrast, the PH does not have the financial incentive for improved health and may benefit from future admissions or visits. Arguably, a hospital system that benefits from future visits may accidentally incentivize poorer outcomes.

Managing hypertension can be stressful and affect milk production. Women with hypertension must be taught how to manage their stress levels and prioritize self-care [33], such as getting adequate sleep, eating a healthy diet, including optimizing magnesium intake [34], and engaging in stress-reducing activities. Overall, hypertension does not necessarily prevent breastfeeding. However, women with hypertension should work closely with healthcare providers to manage their condition and monitor any potential impact on breastfeeding. With proper management, most women with hypertension can successfully breastfeed.

Hospital systems resembling the PH and MH styles can benefit from the initiation of improved metrics assessing breastfeeding initiation and outcomes. If this metric were incentivized, it would likely gain the attention needed to improve breastfeeding rates and outcomes. Providers have little to no training in the effects of various medications on human milk supply, which further illustrates the difficulty of supporting breastfeeding intention. High-risk patients are often incorrectly instructed to “pump-and-dump” based on outdated practices, which complicates and discourages breastfeeding in patients required to take medication for a variety of reasons, given the increased likelihood of morbidities in this population. Standardizing breastfeeding metrics and recommendations and improving provider education could improve outcomes in varied hospital systems. The significant difference in this study’s results encourages further research into hospital system improvements that could make a large-scale difference in breastfeeding outcomes.

## 5. Conclusions

It is vital for pregnant women to receive regular prenatal care and to monitor for signs of an HDP. If an HDP is detected, prompt treatment can help manage the symptoms and prevent serious complications. While often presenting initially as swollen extremities, particularly the feet and ankles, pregnant women afflicted with HDPs can also develop shortness of breath, blurred vision, headaches, and seizures, and, ultimately, die [35]. HDPs are the second most common cause of direct maternal death during pregnancy [36]. Moreover, they are responsible for over one-third of preventable maternal deaths [37]. While educational interventions [38], nutrition education [39], and at-home blood pressure monitoring [40] have been suggested to reduce the adverse outcomes of HDPs, they continue to be a public health concern. Due to the severity of morbidity and mortality, these diseases are an excellent tool to assess two different hospital approaches, the civilian versus military designs, and determine if one system may provide benefits over another.

In this study, the military hospital had significantly higher rates of breastfeeding retention, which should, by all commonly accepted breastfeeding standards, lead to improvements in morbidity and mortality outcomes. While one hospital is not a full representative of a system, it is arguably the single most important detail when comparing the drastic difference in outcomes between these two facilities. If education and preventative medicine metrics can be used to improve civilian systems, it could provide the traction needed to see nationwide breastfeeding improvements.

## Figures and Tables

**Table 1:** Categories of hypertensive disorders of pregnancy by hospital location.

		Chronic Hypertension % (*n*)	Gestational Hypertension % (*n*)	Preeclampsia/ Eclampsia % (*n*)	Total (*n*)
Hospital	PH	2.1% (11) *	78.6% (416) *	19.3% (102) *	529
	MH	21.1% (39) *	44.3% (82) *	34.6% 64 *	185
Total		7% (50)	69.7% (498)	23.3% (166)	714

* *p* = 0.001.

**Table 2:** Infant maturity by hospital location and hypertension category.

		Preterm % (*n*)	Chronic Hypertension % (*n*)	Term % (*n*)	Late Term % (*n*)	Total (*n*)
Hospital	PH	178 (33.7%)	195 (37%)	147 (27.8%)	9 (1.7%) b	529
	MH	7 (3.8%)	114 (61%)	60 (32.4%)	4 (2.2%) b	185
Total			185 (25.9%)	309 (43.3%)	207 (28.9%)	13 (1.8%)

*p* < 0.001 b = Not statistically significant.

**Table 3:** Infant maturity by hospital location, hypertension category, and feeding modality at discharge.

Feeding Modality at Discharge			Diagnosis of			Total
			Chronic Hypertension	Gestational Hypertension	Preeclampsia/Eclampsia	
MF	Hospital Location	Private	1	64	8	73
		Military	6	21	16	43
	Total		7	85	24	116
FF	Hospital Location	Private	5	135	21	161
		Military	2	11	9	22
	Total		7	146	30	183
BF	Hospital Location	Private	5	217	73	295
		Military	31	50	39	120
	Total		36	267	112	415
Total	Hospital Location	Private	11	416	102	529
		Military	39	82	64	185
	Total		50	498	166	714

## Data Availability

Data supporting the reported results are stored at the University of Texas in J.F.’s possession in encrypted files and may be shared upon formal request from the authors.
